# Magnesium sulfate reduces the rocuronium dose needed for satisfactory double lumen tube placement conditions in patients with myasthenia gravis

**DOI:** 10.1186/s12871-019-0841-4

**Published:** 2019-08-31

**Authors:** Shoujun Fei, Hengfu Xia, Xiaowei Chen, Dazhi Pang, Xuebing Xu

**Affiliations:** 1 Department of Anaesthesiology, The University of Hong Kong - Shenzhen Hospital, Shenzhen, China; 2 Department of Thoracic surgery, The University of Hong Kong - Shenzhen Hospital, Shenzhen, China

**Keywords:** Magnesium sulfate, Double lumen tube, Myasthenia gravis, Intubation, Rocuronium

## Abstract

**Background:**

Using a minimum dose of neuromuscular blockade (NMB) to achieve intubation condition is one of the goals in anaesthesia management of patients with myasthenia gravis (MG) for thoracoscopic (VATS) thymectomy. However, tracheal intubation with double lumen tube (DLT) could be challenging if intubation condition is not optimal. This double-blind randomised controlled study was designed to investigate whether magnesium sulfate would reduce the rocuronium dose needed for DLT intubation and improve the DLT placement condition for patients with MG who were scheduled for video-assisted thoracoscopic (VATS) thymectomy.

**Methods:**

Recruited patients were randomly assigned to receive magnesium sulfate 60 mg.kg^− 1^ or normal saline (control) prior to the administration of NMB. Titrating dose of rocuronium was administered to achieve train of four (TOF) ratio less than 10% before DLT intubation. The primary outcome was the rocuronium dose required to achieve TOF ratio less than 10%. The secondary outcome was intubation condition for DLT placement.

**Results:**

Twenty-three patients had received magnesium sulfate and 22 patients had received normal saline before rocuronium administration. The required rocuronium dose [mean (standard deviation)] were 0.10 (0.05) mg.kg^− 1^ and 0.28(0.17) mg.kg^− 1^ in patients who had magnesium sulfate and normal saline respectively(*P* < 0.0001). With a similar depth of neuromuscular blockade and depth of anaesthesia, 100% of patients in the magnesium sulfate group and 72.7% of patients in the control group showed excellent intubation condition (*P* = 0.027) respectively. The patients in both groups had similar emergence characteristics.

**Conclusions:**

Magnesium sulfate is associated with a decrease in rocuronium requirement for an optimal DLT intubation condition in patients with MG for VATS thymectomy.

**Trial registration:**

Clinical Trial Registry of China (http://www.chictr.org.cn) identifier: ChiCTR-1800017696, retrospectively registered on August 10, 2018.

## Background

Myasthenia Gravis (MG) is a chronic neuromuscular disease characterized by skeletal muscle weakness. Maximal thymectomy is one of the treatment options recommended for all patients who have mild to moderate muscle weakness due to MG [1]. There are several different approaches for maximal thymectomy, of which right sided video-assisted thoracoscopic (VATS) thymectomy is gaining popularity [2]. Patients with MG are highly sensitive to nondepolarizing neuromuscular blockers [3]. The use of neuromuscular blocker (NMB) in these patients is always controversial because its use is associated with increased risk of failed extubation and postoperative respiratory failure [3, 4]. Moreover, perioperative stress may also lead to exacerbation of MG and postoperative respiratory failure [4–6].

Rocuronium combined with its specific antagonist, sugammadex, may be a good option for muscle relaxation management for patients with MG, which could largely decrease the risk of failed extubation and postoperative respiratory failure [7–9]. Unfortunately, high price plus other factors, such as marketing promotion and the policy limitation on local Medicare reimbursement drug, confine the clinical use of sugammadex [10], and also some patients may be contraindicated to sugammadex for anaphylaxis reason [11]. Although it is possible to perform intubation without NMB, the intubation condition is often unsatisfactory and this may increase the incidence of upper airway injury [12]. In our institution, we routinely titrate small doses of non-depolarizing NMB with train of four (TOF) ratio monitoring. Intubation is performed when the TOF ratio is less than 10%. For patients undergoing VATS, a satisfactory intubation condition is especially important because the placement of double lumen tube (DLT) demands much better intubation condition when compared with single lumen tube.

Magnesium sulfate is an agent with analgesic, anaesthetic and NMB effects [13, 14]. Previous researches have shown that after the administration of rocuronium, the time required to achieve 95% suppression of TOF was shortened if patients were pretreatment with magnesium sulfate [15]. This investigation was designed to evaluate whether the use of magnesium sulfate could decrease the dose of rocuronium and improve the DLT intubation condition in patients with MG. The hypothesis is that the use of magnesium sulfate prior to the administration of rocuronium would reduce the rocuronium dose required to achieve a satisfactory intubation condition as monitored by TOF ratio.

## Patients and methods

The study was approved by the Hospital Institutional Review Board and registered with the Clinical Trial Registry of China (ChiCTR-1800017696). This manuscript adheres to the applicable 2010 CONsolidated Standards of Reporting Trials (CONSORT) guidelines. Patients who were diagnosed with MG of grade I~II of Osserman classification and scheduled to undergo right sided VATS thymectomy were enrolled in this study between May 2016 and May 2018 after written informed consent. The diagnosis was confirmed by the presence of circulating antibody to the acetylcholine receptor, typical clinical and laboratory findings including ptosis, diplopia, limb weakness, and a decremental conduction response on electrical stimulation of the nerve supply to the deltoid muscle. Exclusion criteria were suspected difficult intubation, body mass index > 30 kg.m^− 2^, age less than 18 or over 60 years, hepatic or renal dysfunction, cardiovascular dysfunction, neurologic disorder, operating time greater than 4 hours, intraoperative blood loss over 1000 ml, history of chronic pulmonary disease, chronic medication with calcium channel blocker or magnesium, and coexisting autoimmune diseases including hyperthyroidism, rheumatoid arthritis, scleroderma or lupus.

The selected patients were randomly assigned to receive magnesium sulfate or normal saline (control). A random sequence was kept within a sealed opaque envelope by a research assistant not involved in this study. On the morning of the surgery, the assistant opened a sealed envelope and prepared the study drug which includes magnesium sulfate (60 mg.kg^− 1^ in 50 ml normal saline) or 50 ml normal saline according to the group allocation. The attending anaesthetists were blinded to the patient’s allocation.

Patients would take their usual dose of anticholinesterase medication and/or steroid on the day of surgery. No premedication was given to the patients. Anaesthesia monitor placed before induction of anaesthesia included pulse oximetry (SpO_2)_, electrocardiograohy (ECG), noninvasive blood pressure (NIBP), arterial blood pressure and bispectral index (BIS™ sensor; Medtronic, Minneapolis, MN, USA). Bispectral index was recorded using BISx Power Link™ by Philips Medical Systems (Royal Philips Electronics, Eindhoven, The Netherlands). The invasive arterial blood pressure monitor was achieved by left radial artery cannulation under local anaesthesia. An 18G peripheral venous access was placed after performing perioperative checklist was performed with the surgical and nursing team. Patients’ vital signs were recorded and retrieved from the automatic anaesthesia information system.

Anaesthesia was induced with 4 μg.kg^− 1^ fentanyl and propofol with target controlled infusion (TCI) using Marsh model (Fresenius Kabi AG, Germany). Propofol TCI was commenced at an effect-site concentration (Ce) of 2 μg.ml^− 1^ and titrated to achieve unconsciousness with BIS at 40–60. The patient was ventilated with face mask to maintain the end tidal carbon dioxide between 30 and 45 mmHg. After the depth of anaesthesia was stable with BIS kept between 40 and 60 for 10 min and a stable Ce of propofol, the electrical stimulation of neuromuscular monitor was applied at the ulnar nerve for the contraction of the adductor pollicis muscle using TOF at amplitude 50 mA with time interval at 20 s (ISx Power Link™ by Philips Medical Systems). After the baseline TOF ratio was obtained, the study drug (magnesium sulfate or normal saline) was given over 5 min. Another TOF ratio was obtained after study drug infusion was completed. If the TOF ratio was above 10%, a repeated dose of 0.05 mg.kg^− 1^ rocuronium was given every 3 min until the TOF ratio was less than 10%. The patient was intubated with DLT using video laryngoscopy when TOF ratio was less than 10%. The intubation was performed by an experienced anaesthetist. If the tracheal intubation was not accomplished within 20 s, it was recorded as a failed attempt. Mean blood pressure (MAP) and heart rate (HR) were recorded 1 minute before intubation (Pre-intubation), and 3 minutes after intubation (Post-intubation).

Anaesthesia was maintained with propofol using TCI to keep BIS at 40–60. Analgesia was achieved with local anaesthesia infiltration using 0.5% ropivacaine 10 ml before skin incision. Remifentanil TCI infusion at 2~4 ng.ml^− 1^ was used intraoperatively. No further dose of NMB was administered after induction. Intravenous 40 mg parecoxib was administered at 15–30 min before the end of operation for postoperative pain, and 4 mg ondansetron plus 5 mg dexamethasone was also used for prevention of postoperative nausea and vomiting (PONV). When the surgery was completed, neostigmine 0.05 mg.kg^− 1^(along with atropine) and calcium chloride 1 g were given to the patient if the TOF ratio was less than 90% or the anaesthetist was not satisfied with the recovery of the respiratory function. During the operation, 5 ml.kg^− 1^.h^− 1^ Ringer’s lactate solution was given and no Foley catheter was used. Phenylephrine was used to maintain blood pressure when necessary. All thymectomy were conducted with right sided VATS with three ports. Before closing the final incision for VATS, negative suction of chest cavity combined with lung recruitment manoeuvers was employed to re-expand the right collapsed lung, and no postoperative chest drain was used.

The primary outcome of this study was the cumulative rocuronium dose needed to achieve a TOF ratio less than 10% before tracheal intubation. The secondary outcome was intubation condition for DLT placement. The DLT intubation condition quality was evaluated based on Copenhagen Consensus Conference scoring system which includes the ease of laryngoscopy, vocal cord position and/or movement and response to intubation (cough or diaphragmatic movement). Intubation condition was classified as excellent, fair or difficult [16, 17]. Other secondary outcomes included were laryngoscopy and intubation induced MAP and HR changes (Post-intubation vs. Pre-intubation), propofol concentration during tracheal intubation, time from the last dose of rocuronium before intubation to TOF ratio 90% recovery, the time to extubation after completion of surgery. Postoperative data collection included the visual analogue score (VAS) for pain, postoperative Riker sedation and agitation scales and PONV status. We classified the patients’ postoperative pain intensity as no pain, mild pain, moderate pain and severe pain with VAS of 0–4 mm, 5–44 mm, 45–74 mm, and 75–100 mm respectively [18]. Utilizing the Riker sedation and agitation scales, we further divided the patients into three categories according to the scale: over sedated (scales 1–2), non-agitated (scales 3–4) and agitated (scales 5–7) [19, 20].

### Statistical analysis

The sample size estimation was based on the primary outcome (cumulative rocuronium dose used for intubation) in our pilot study. The mean difference of the initial rocuronium dose between the magnesium sulfate group and the normal saline group was 0.14 mg.kg^− 1^ with a pooled variance (SD) of 0.11. To obtain an alpha value of 0.05 and a test power of 80%, about 12 subjects were needed in each group. Anticipating about 20% dropout rate, more than 15 subjects were needed in each group. Finally, we planned to recruit 30 patients per group.

Continuous variables presented as mean (SD) or number (%) and were compared by Student t-test. Categorical variables are presented as the number of patients and were compared by Chi-square test or Fisher’s exact test. A *P* value < 0.05 was considered significant. Data analysis was accomplished using MedCalc for Windows, version 11.4.2.0 (MedCalc Software, Mariakerke, Belgium).

## Results

**Fig. 1:**
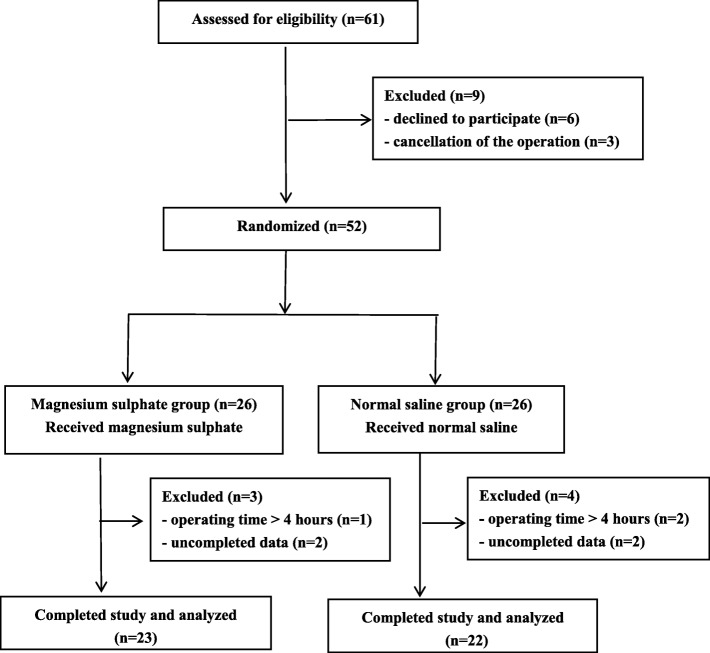
Consort flowchart

Sixty-one eligible patients were approached to participate in the study. Nine patients refused to participate, two patients in each group were excluded for incomplete observational data collection. One patient in the magnesium sulfate group and two patients in the control group were excluded for unexpected prolong operating time (greater than 4 hours). Finally, 23 patients who received magnesium sulfate and 22 patients who received normal saline were included in the analysis (Fig. 1).

Patients’ demographic data including gender, age, BMI, Osserman classification and length of MG history in both groups showed no difference (Table 1). All intubation procedures were achieved with one attempt. No difference was found between the two groups in operating time (Table 1). Pretreatment with magnesium sulfate was associated with a significantly smaller dose of rocuronium required to meet the target depth of neuromuscular blockade (Table 2). Moreover, there were two patients did not require rocuronium for intubation in the magnesium sulfate group. The overall intubation condition was significantly better in the patients who had magnesium sulfate (Table 2). Moreover, significantly fewer patients who had received magnesium sulfate had postoperative agitation (Table 2). Tracheal intubation induced a significant increase of MAP and HR in the control group, but not in the magnesium sulfate group (Table 3). There was no difference in other secondary outcomes and those include the Ce of propofol at intubation, the rate of postoperative neostigmine medication, time of TOF ratio 90% recovery from the last dose of rocuronium before intubation, the time to extubation and postoperative pain intensity (Table 2). There was no reported PONV in post-anaesthesia care unit (PACU).

**Table 1:** Patient characteristics. Data are presented as mean (standard deviation) or number of patients

	Magnesium sulfate group (*n* = 23)	Normal saline group(*n* = 22)	*P*
Age (year)	34.4 (11.3)	30.3 (9.0)	0.185
Body weight	55.8 (8.0)	60.5 (11.0)	0.111
BMI (kg.m^−2^)	21.7 (2.0)	22.2 (3.0)	0.577
Sex (Male/Female)	4/19	7/15	0.260
MG history ≥6 years/< 6 years	6/17	8/14	0.457
Osserman stage(I/IIa/IIb)	6/11/6	8/6/8	0.364
Operating time (minutes)	110.1 (31.6)	117.7 (24.4)	0.370

**Table 2:** Anaesthesia and emergence data. Data are presented as mean (standard deviation) or number of patients

	Magnesium sulfate group (*n* = 23)	Normal saline group(*n* = 22)	*P*
Rocuronium dosage (mg.kg^− 1^)	0.10 (0.05)	0.28(0.17)	< 0.0001*
Intubation condition score (excellent/fair/poor)	23/0/0	16/5/1	0.027*
Propofol concentration when intubation (μg.ml^−1^)	3.15(0.36)	3.37(0.91)	0.267
Time of TOF ratio 90% recovery from the last dose of rocuronium before intubation (minutes)	50.5 (42.4)	47.2(42.2)	0.881
Time of extubation time from the end of operation (minutes)	9.4(5.6)	10.5(6.8)	0.561
Postoperative neostigmine medication (no/yes)	17/6	15/7	0.672
Postoperative pain intensity (free/mild/moderate)	15/7/1	8/6/6	0.056
Riker sedation and agitation scale in PACU (non-agitated/agitated)	23/0	16/6	0.009*

Intubation condition score was evaluated based on Copenhagen Consensus Conference scoring system which includes the ease of laryngoscopy, vocal cord position and/or movement and response to intubation (cough or diaphragmatic movement) [16, 17]

Excellent: all qualities are excellent; Good: all qualities are either excellent or good; Poor: difficult of laryngoscopy, vocal cords closed or vigorous/sustained response to intubation

*P value < 0.05 was considered significant

**Table 3:** MAP and HR 1 minute before intubation (Pre-intubation) and 3 minutes after intubation (Post-intubation). Data are presented as mean (standard deviation)

	MAP (mmHg)		HR (bpm)	
	Magnesium sulfate group (*n* = 23)	Normal saline group (*n* = 22)	Magnesium sulfate group (*n* = 23)	Normal saline group (*n* = 22)
Pre-intubation	65.6 (11.7)	69.5 (12.3)	62.0 (13.5)	59.0 (9.2)
Post-intubation	69.3 (12.3)	86.2 (7.42)	66.8 (12.7)	76.4 (17.1)
*P*	0.309	< 0.0001*	0.218	0.001*

*MAP* Mean arterial blood pressure, *HR* heart rate, Pre-intubation: 1 minute before starting to intubation; Post-intubation: 3 minutes after intubation

*P value < 0.05 was considered significant

After the study drug was given, the TOF ratio of patients in magnesium sulfate group dropped from 95.7%(10.5%) to 77.2%(29.2%), which showed a significant decrease (*P* = 0.0095); whereas the TOF ratio of patients in the control group was quite stable (*P* = 0.211), changed from 94.7% (12.2%)to 95.9% (9.6%). The MAP and HR showed no significant change during and after magnesium sulfate infusion.

After the operation, there were six patients in the magnesium sulfate group and seven patients in the control group respectively administered neostigmine for reversal of NMBA. There was no difference in the rate of neostigmine medication. All the patients were extubated in the operating room and transferred to ward after recovery in the PACU. There was no incidence of postoperative myasthenic crisis and re-intubation in any patients.

## Discussion

Patients with myasthenia gravis are extremely sensitive to nondepolarizing NMBs [3, 7]. A very small dose of NMB and residual neuromuscular blockade effect may result in respiratory distress or loss of airway protection during emergence from anaesthesia. As a result, some anaesthetists prefer to avoid NMB, whereas intubation without NMBs was reported to increase the risk of difficult tracheal intubation and intubation-related complications [4, 5].

Sugammadex could help to solve the problem of inadequate muscle relaxation and residual neuromuscular blockade. While sugammadex is not widely used for MG patients for many reasons. In mainland China, less than 10% of the hospital have stocked sugammadex because it is expensive and not included in the basic Medicare reimbursement drug inventory. Therefore, using a minimal dose of intermediate-acting NMB is still quite a common choice of tracheal intubation for patients with MG [7, 8]. In this study, we have revealed that the pre-administration of magnesium sulfate at 60 mg.kg^− 1^ is associated with a significant decrease in rocuronium requirement with improving tracheal intubation condition for DLT placement.

Magnesium possesses an inhibitory effect on neuromuscular transmission and caused a decrease in muscle fibre membrane excitability. It decreases pre-junctional release of acetylcholine from the motor nerve terminal by decreasing the calcium conductance of presynaptic voltage-dependent calcium channels, and it also reduced the sensitivity of the endplate to acetylcholine [21, 22]. Magnesium shows significant neuromuscular blockade at high plasma concentrations (5 to 10 mM), and also at lower concentrations (≥1 mM) in the presence of neuromuscular-blocking agents [21, 22]. Several studies had shown that magnesium sulfate could decrease the amount of rocuronium required to maintain adequate neuromuscular blockade during different types of surgery [23, 24]. In our study, magnesium sulfate pretreatment has led to a reduction of TOF ratio from 96 to 77%, and this has resulted in a significant decrease of the rocuronium dose needed to achieve the target TOF ratio.

Interestingly, although patients from the two groups had a similar degree of neuromuscular block, during DLT placement the patients in the magnesium sulfate group had better tracheal intubation condition with less hemodynamic change caused by laryngoscopy and tracheal intubation. This may be related to the anti-noxious stimulation effects of magnesium sulfate on the laryngoscopy and tracheal intubation [25]. The mechanism of this action is proved to be inhibition of catecholamine release from adrenal medulla and adrenergic nerve endings [13]. Our results are similar to that of other investigations for single lumen tube placement [26, 27].

Though some research had shown that magnesium sulfate administration is associated with a decrease in the intravenous and inhale anaesthetic dose for induction [13, 28], it is not shown in this study. This discordance is possibly related to the small sample size in our study, and magnesium sulfate might exert more enhancement effects on neuromuscular blocker than that on anaesthetic agents.

Though there are two meta-analyses which independently concluded that perioperative systemic magnesium sulfate administration could decrease the postoperative pain scores and opioid consumption [29, 30], we have not observed the significant analgesic benefit of magnesium sulfate in this study. This could be related to the lack of continuous infusion of magnesium sulfate during the VATS procedure. Moreover, the overall pain intensity is minimal with the multimodal analgesic regimen, therefore, it would be difficult to detect any difference between the two groups. Significantly more patients in the magnesium sulfate group were comfortable with less agitation and this is consistent with Abdulatif’s finding that magnesium sulfate decreased the incidence and severity of emergence agitation in children undergoing adenotonsillectomy [31].

When selecting a drug for a specific purpose, you should consider the balance between its benefits and associated side-effects. The side effects of magnesium are pain on injection site, hypotension, bradycardia, nausea/vomiting and muscle weakness. Magnesium sulfate (50–60 mg.kg^− 1^) was not associated with serious complications in previous studies [29, 30], and respiratory depression caused by muscle weakness, the most severe adverse effect of a large dose of magnesium, did not occur in our study. According to clinical pharmacology of magnesium sulfate applied to obstetrics, the estimated magnesium concentration is about 2–3 mmol. L^− 1^ after administration of magnesium sulfate 60 mg.kg^− 1^, which is less than the concentration resulting in muscle weakness(3.8–5 mmol. L^− 1^) and respiratory depression(6.3–7.1 mmol. L^− 1^) [32]. In the present study, the MAP and HR were stable during and after magnesium sulfate infusion. Combined with the advantage of magnesium sulfate on the decrease of rocuronium dose and improvement of intubation condition for DLT, we suggested that intravenous magnesium sulfate might be an appropriate adjuvant for DLT intubation in patients with MG. Although magnesium sulfate had been extensively investigated as an adjuvant for neuromuscular blocker, this is the first study to describe the benefits of magnesium sulfate on DLT placement in patients with MG. It is important to note that the time of 90% recovery of TOF ratio was about 50 min in our study, our technique may not be applicable for short surgical procedure.

There are two main limitations of this study. The first is that the patients enrolled in our study were classified Osserman I~II and aged less than 60 years. The effects of magnesium sulfate on these patients may be different from that on patients with more severe MG and in patients who are older than 60, therefore our conclusion may not be applied in these patients. The second limitation is related to the titration method of rocuronium, the dose of total rocuronium in the control group was larger than that of the magnesium sulfate group; thus the duration of rocuronium administration in the control group was longer than that of the magnesium sulfate group. This duration difference resulted in a difference in total anaesthesia time and could be a confounding factor for emergence characteristics of the two groups.

In conclusion, the use of magnesium sulfate is associated with a decrease in the rocuronium dose for an optimal DLT tracheal intubation condition in patients with MG.

## Data Availability

The datasets used and/or analyzed during the current study are available from the corresponding author on reasonable request. The datasets used are also available from Clinical Trial Registry of China (http://www.chictr.org.cn/index.aspx).
